# Immunohistochemical Studies of Age-Related Changes in Cell Proliferation and Angiogenesis during the Healing of Acetic Acid-Induced Gastric Ulcers in Rats

**DOI:** 10.1155/2020/3506207

**Published:** 2020-05-31

**Authors:** A. Folorunsho Ajayi, S. Babafemi Olaleye

**Affiliations:** ^1^Department of Physiology, College of Medicine, University of Ibadan, Ibadan, Nigeria; ^2^Department of Physiology, Ladoke Akintola University of Technology, Ogbomoso, Nigeria

## Abstract

Cell proliferation and angiogenesis are of utmost importance for healing to take place. The KI67 and EGFR proteins are markers of cell proliferation, while CD31 and factor VIII are markers of angiogenesis. To elucidate the mechanism responsible for delayed healing of the gastric injury in old age, we analyzed the expression of these markers in rats of different months during the healing of an acetic acid-induced gastric ulcer. Male Wistar rats (aged 3, 6, 12, and 18 months) divided into four groups, according to their ages, formed the experimental animals. Stomach tissue samples were collected on days 3, 7, 14, and 21 after induction for assessment of ulcer healing. The area of gastric mucosa healed was inversely proportional to age. The expression of markers of proliferation (KI67 and EGFR) and angiogenesis (factor VIII and CD31) decreased significantly (*p* < 0.05) in older rats when compared with younger ones (3 months > six months > 12 months > 18 months) on days 7, 14, and 21 after induction of gastric ulcer. This study revealed that the slower gastric ulcer healing rate in older rats might be due to reduced epithelial cell proliferation and angiogenic activities.

## 1. Introduction

Gastric ulcer healing is a genetically determined restoration process which includes inflammation, cell proliferation, reepithelialization, granulation tissue formation, and angiogenesis. The interaction between different cells and the matrix tissue remodelling results in scar formation [1, 2]. Cytokines control the events above, with the help of various growth factors (epidermal growth factor (EGF), platelet-derived growth factor (PDGF), keratinocyte growth factor (KGF), hepatocyte growth factor (HGF), transforming growth factor beta (TGF-*β*), and vascular endothelial growth factor (VEGF)). The formation of new blood vessels and transcription factors activated by tissue injury in spatial and temporal manner coordinate the healing process [3–5].

The stomach lining can combat damage decreases with age as a result of changes in gastroprotection and decreased mucosal blood flow [6]. Ageing is usually associated with age-linked changes in motor function of the different processes involving the digestive tract, such as transit time and gastric emptying [7, 8]. The occurrence of digestive diseases increases with age; this corresponds with age-related changes in the structural and functional integrity of the gastrointestinal tract. These changes include mucosa, muscular coat, and blood flow [9–12].

Animal models employed in the study of the role of ageing in gastric mucosal proliferation and regeneration are many. Majumdar and his coworker have reported the higher vulnerability of older rats to acute gastric injury induced with hypertonic saline solution. That proliferative response is more significant in younger rats' gastric mucosa compared to the older ones [13]. Studies on the mechanism of action have shown that ageing is related to reduced regenerative potentials of stomach mucosa damaged by hypertonic saline. The reduction in mucosa regeneration due to age is as a consequence of decreased expression of transforming growth factor-alpha. Growth factor receptor such as tyrosine kinases also reduced in expression [14, 15] in the stomach mucosa of old rats.

The ability of the stomach mucosa in healthy adult to repair spontaneously after a sudden assault is outstanding. However, the mechanism regulating this repair, as well as the potential modulating effects of ageing, is poorly understood. The modulating fact of ageing on repair processes of gastric mucosa after an injury such as inflammation, cell proliferation, reepithelialization granulation tissue formation, and angiogenesis with regulatory factors, which include growth factors and apoptosis, remains an area of research interest. Therefore, the influence of age in the expression of some molecular factors involved in the healing process of the experimental ulcer was investigated to elucidate the mechanism by which age influences gastric ulcer healing.

## 2. Methods

### 2.1. Experimental Animals

**Figure 1:**
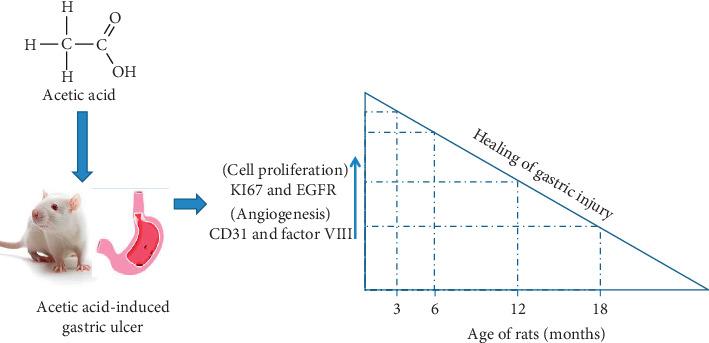
Graphical abstract.

Eighty male Wistar rats (ages 3, 6, 12, and 18 months) used in the study were bred specially to ascertain their ages, with the parent stock acquired from the Faculty of Basic Medical Sciences Animal House, University of Ibadan, Nigeria. The rats were taken care of in standard cages and were given commercial rat pellets (Ladokun Feeds Limited, Nigeria) with access to water *ad libitum*. The rats were, after that, separated into four groups according to their ages; each group containing 20 rats. All animals got humane care with the procedures conforming to the guiding principles for research involving animals, recommended by the Declaration of Helsinki together with the Guiding principles in the care and use of animals [16]. The research protocol was submitted to the Ministry of Health, Oyo State, Nigeria, and letter of ethical permission for the conduct of the research was given with reference number: AD 13/479/452.  Figure 1 illustrates the graphical representation of the experimental design.

### 2.2. Acetic Acid-Induced Ulceration

Gastric ulcers were induced using acetic acid following the method described by Tsukimi and Okabe [17] with a little modification. The rats were deprived of feed for 24 hours. The stomach of each rat was exposed under anaesthesia (a mixture of xylazine (0.0005 ml/g b.w.) and ketamine (0.0015 ml/g b.w.)) by performing laparotomy through a midline epigastric incision. The stomach glandular walls were clamped with a pair of eye forceps rings, and 0.2 ml of an acetic acid mixture (40% v/v distilled water) was injected into the intraluminal secretory area of the stomach and withdrawn after 45 seconds. The stomach surface was then bathed with normal saline to prevent tissue adherence, the abdomen were sutured, and rats were allowed to recover. All the animals were fed normally throughout the experimental period with samples collected on days 3, 7, 14, and 21 after induction. We obtain *n* = 5, where *n* is the number of rats involved in a particular experiment.

### 2.3. Macroscopic Evaluation of Ulcers

Gastric ulcer areas in mm^2^ were determined on days 3, 7, 14, and 21 after induction of ulcer, with five rats taken from each group each day. The rats were sacrificed by cervical dislocation, and the stomach was removed, with the greater curvature opened, washed with normal saline, and pinned on a corkboard. The macroscopic ulcer area was after that measured using a 2x magnification hand lens, then measured, and calculated using the collection of guiding principles of drug administration of the Ministry of Health Beijing, 1993, as reported by Salami et al. [18] using the following formula:

(1) $$\begin{array}{c} S=\pi \left(\frac{d1}{2}\right)\times \left(\frac{d2}{2}\right), \end{array}$$

where *S* stands for the ulcerated area (mm^2^), *d*1 represents the longest longitudinal diameter of the ulcer, and *d*2 is the longest transverse diameter of the ulcer.

The calculation of percentage area of ulcer healed was done as described by Adeniyi et al. [19]:

(2) $$\begin{array}{c} \text{Percentage area healed on day }7=\frac{\text{area of an ulcer on day}3-\text{the area of an ulcer on day }7}{\text{area of an ulcer on day }3}\times 100,\\ \text{Percentage area healed on day }14=\frac{\text{area of an ulcer on day}3-\text{the area of an ulcer on day }14}{\text{area of an ulcer on day }3}\times 100,\\ \text{Percentage area healed on day }21=\frac{\text{area of an ulcer on day }3-\text{the area of an ulcer on day }21}{\text{area of an ulcer on day }3}\times 100. \end{array}$$

### 2.4. Sample Processing and Immunohistochemical Analysis

Histological analysis of the sample was carried out as described by Ogihara and Okabe [20]. In brief, small sections of the stomach were taken from two distinct areas of the stomach body (from each rat) and placed in 10% formalin for histological preparations. The extracted stomach tissue was fixed, cut into five *μ*m sections, and stained with hematoxylin and eosin. Studies involving immunohistochemistry were done using the avidin*-*biotin peroxidase complex (ABC) in immunoperoxidase techniques by Hsu et al. [21]. A section of 3 microns of thickness was cut from the paraffin-embedded tissue block and allowed to warm on a hot plate for one hour at 70°C, followed by deparaffinization of sections in xylene and rehydrating through graded ethanol to distilled water (i.e., 100%, 95%, 90%, and 70% H_2_0). For antigen retrieval, sections were placed in prewarmed “Target Unmasking Fluid” citric acid solution with pH 6.0 (1 : 10 dilution) in a microwave at power 100 for 15 minutes. Then the section is equilibrated by gently displacing in hot citric acid with running tap water for 3 minutes. The peroxidase in tissue was blocked using peroxidase block (0.3%H_2_0_2_) for 15 minutes and then washed with excess water and stabilized with phosphate-buffered saline (PBS) (in 1-litre distilled water, dissolve 8 g sodium chloride, 1.44 g disodium hydrogen phosphate, 0.24 g potassium dihydrogen phosphate, and 0.2 g potassium chloride. pH adjusted to 7.4 using HCI) mixed with tween 20 for 2 minutes, and the nonspecific binding proteins were blocked with egg. Avidin protein was blocked in a humidified chamber for 15 minutes and, after that, removed gently by washing for 2 minutes with PBS. Then, the sections were incubated with primary antibodies: anti-rat KI67, EGFR, CD31, and Factor VIII (Novacastro laboratories, United Kingdom) in a humid chamber for 45 minutes, after which it was washed extensively with PBS for 3 minutes. The secondary biotinylated antibody was then used for incubation for 45 minutes at room temperature followed by washing in PBS thrice. A polymer was then added to initiate polymerization, by incubating with streptavidin horseradish peroxidase system (Dako AS, Denmark) for 15 minutes, and then washed twice with PBS. Adding peroxidase substrate DAB for 15 minutes and brown precipitate developed, this indicates positive reaction, which was then washed with water and counterstained for 2 minutes with hematoxylin, followed by dehydration in graded ethanol, and then cleared with xylene, followed by mounting with DPX, and examined under a light microscope at magnification ×40.

### 2.5. Labelling Index Calculation from ImmunoRatio Web Application

The evaluation of staining intensity and quantity of cells stained by KI67, EGFR, CD31, and factor VIII proteins were evaluated in correspondence to the ulcerated areas. Brown staining of the cytoplasm and nucleus was viewed under ×40 magnification. Positive control slides prepared from cell known to express the protein and control negative slides were made by skipping the incubation state with the primary antibody. The percentages of positively stained nuclei for KI67, EGFR, CD31, and factor VIII were quantified using ImmunoRatio web application (http://jvsmicroscope.uta.fi/immunoratio/) for Image J (http://imagej.nih.gov/ij/). This application resides on a remote server accessed through the Internet with a web browser. The main features include separating diaminobenzidine-stained (DAB) from hematoxylin-stained regions of the image, calculating the percentage of DAB-stained part over the entire region, which is known as the labelling index and generating a pseudo-coloured image corresponding with the area segmentation [22].

### 2.6. Statistical Analysis

All values are considered as mean ± SEM (standard error of the mean) in this study, and *n* stands for the number of rats used for a particular experiment. The statistical significance of differences among the groups was analyzed using one-way ANOVA. Value with *p* < 0.05 was considered significant.

## 3. Results

### 3.1. Macroscopic Evaluation of Ulcers

On the third day, there was no significant difference (*p* > 0.05) between the ulcer areas of 3- and 6-month-old rats, but the 12- and 18-month-old rats' ulcer areas were significantly different from others. Ulcer areas significantly (*p* < 0.05) decreased in younger rats (3 and 6 months old) compared to older ones (12 and 18 months old) on days 7, 14, and 21 after induction as shown in Table 1.

**Figure 2:**
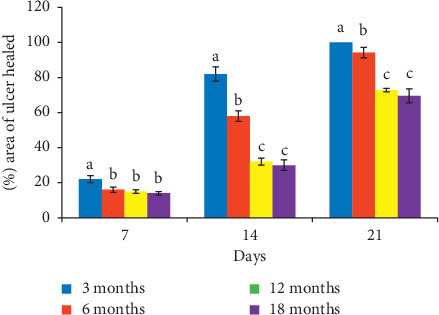
Age-related changes in the area of ulcer healed on days 7, 14, and 21 about day three after induction of ulcer. Bars with letters a, b, and c are statistically different at *p* < 0.05.

Figure 2 shows that the area of gastric mucosa healed in 3-month-old rats was significantly (*p* < 0.05) higher than other age groups on days 7 and 14. On day 21, the three-month-old gastric mucosa healed utterly (100%), while 6-, 12-, and 18-month-old rats had 94.16%, 75.77%, and 69.58% area of mucosa healed, respectively.

### 3.2. Immunohistochemical Studies

#### 3.2.1. Cell Proliferation

**Figure 3:**
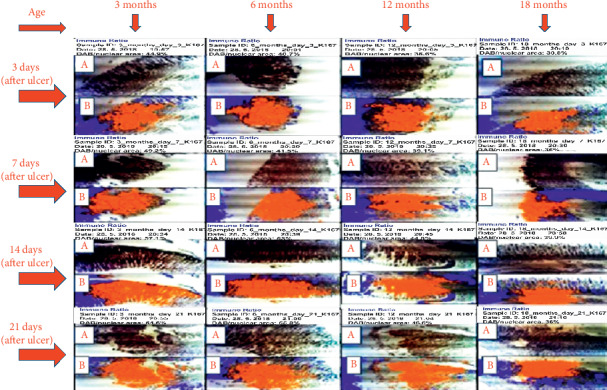
Photomicrograph of expression of KI67 on days 3, 7, 14, and 21 after induction of ulcer. A represents slide of gastric mucosa with original DAB-stained image. B represents slide of gastric mucosa with a pseudo-coloured image produced by ImmunoRatio web application showing staining components from which labelling index ensued.

**Figure 4:**
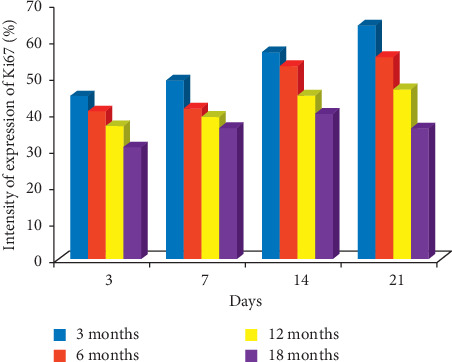
Bar chart indicating the age-related changes in the intensity of expression of KI67 on the gastric mucosa during the healing of gastric ulcer induced with acetic acid.

KI67: on day 14, labelling indices of 57.1%, 53.0%, 44.8%, and 39.9% were obtained for 3-, 6-, 12-, and 18-month-old rats, respectively. On day 21, the labelling indices of 64.6%, 55.8%, 46.6%, and 36.0% were obtained for 3-, 6-, 12-, and 18-month-old rats, respectively; this means that the highest expression of KI67 was seen in 3-month-old rats during healing as shown in Figures 3 and 4.

**Figure 5:**
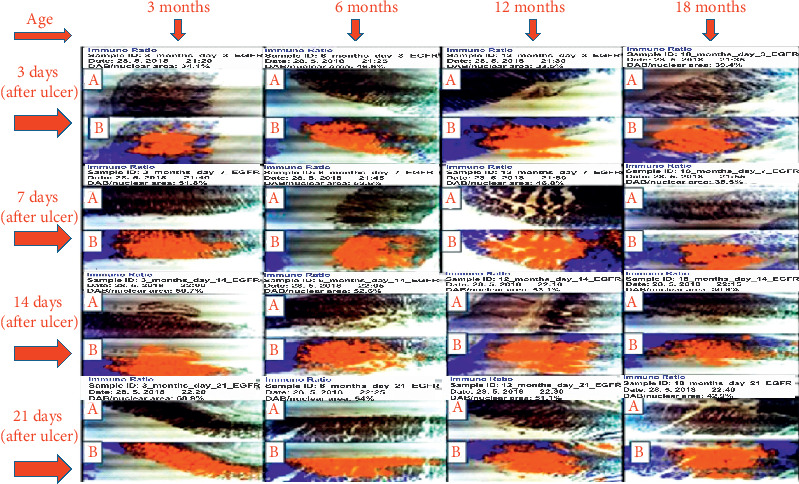
Photomicrograph of expression of EGFR on days 3, 7, 14, and 21 after induction of ulcer. A represents slide of gastric mucosa with original DAB-stained image. B represents slide of gastric mucosa with a pseudo-coloured image produced by ImmunoRatio web application showing staining components from which labelling index ensued.

**Figure 6:**
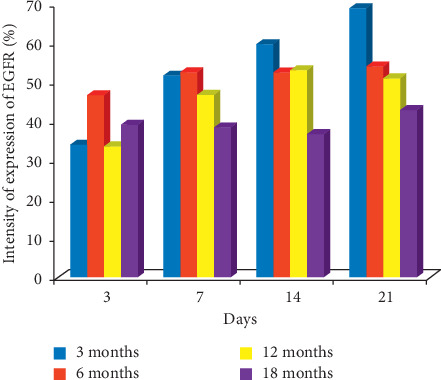
Bar chart indicating the age-related changes in the intensity of expression of EGFR on the gastric mucosa during the healing of gastric ulcer induced with acetic acid.

EGFR: EGFR showed the same pattern of expression recorded for KI67 on days 14 and 21. On day 14, labelling index of 59.7%, 52.6%, 53.1%, and 36.9% ensued for 3-, 6-, 12-, and 18-month-old rats, respectively. On day 21, the labelling index of 68.9%, 54.0%, 51.1%, and 42.9% was obtained for 3-, 6-, 12-, and 18-month-old rats, respectively, which also suggests that the highest expression of EGFR was seen in 3-month-old rats during healing as shown in Figures 5 and 6.

#### 3.2.2. Angiogenesis

**Figure 7:**
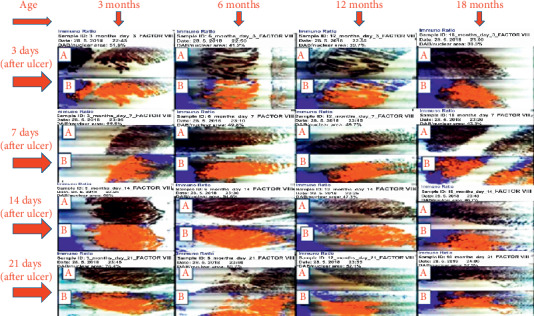
Photomicrograph of expression of factor VIII on days 3, 7, 14, and 21 after induction of ulcer. A represents slide of gastric mucosa with original DAB-stained image. B represents slide of gastric mucosa with a pseudo-coloured image produced by ImmunoRatio web application showing staining components from which labelling index ensued.

**Figure 8:**
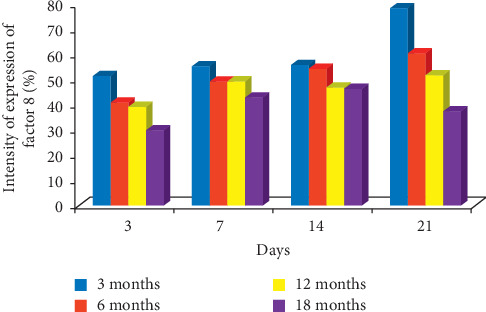
Bar chart indicating the age-related changes in the intensity of expression of factor VIII on the gastric mucosa during the healing of gastric ulcer induced with acetic acid.

Factor VIII: the expression of factor VIII was higher in 3-month-old rats than other age categories on days 3, 7, 14, and 21 after induction of ulcer. On day 14, labelling index of 56.0%, 54.6%, 47.3%, and 46.7% was obtained for 3-, 6-, 12-, and 18-month-old rats, respectively. On day 21, the labelling index of 78.4%, 60.7%, 52.1%, and 37.9% was obtained for 3-, 6-, 12-, and 18-month-old rats, respectively, which also suggests that the highest expression of factor VIII was seen in 3-month-old rats during healing as shown in Figures 7 and 8.

**Figure 9:**
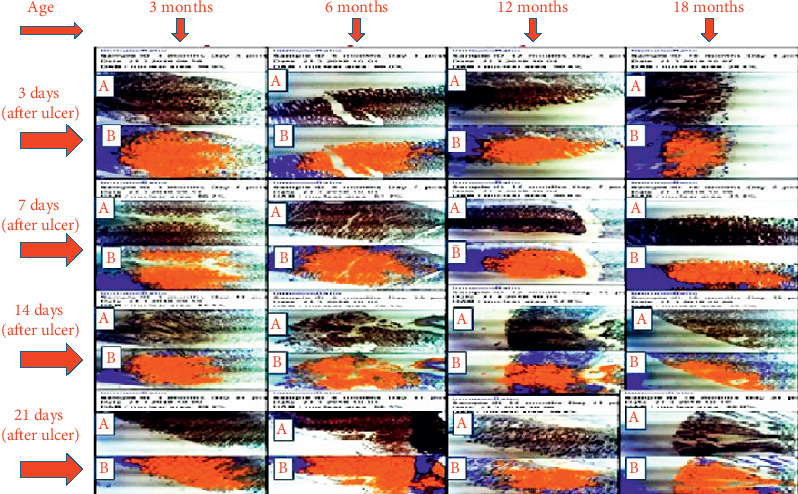
Photomicrograph of expression of CD31 on days 3, 7, 14, and 21 after induction of ulcer. A represents slide of gastric mucosa with original DAB-stained image. B represents slide of gastric mucosa with a pseudo-coloured image produced by ImmunoRatio web application showing staining components from which labelling index ensued.

**Figure 10:**
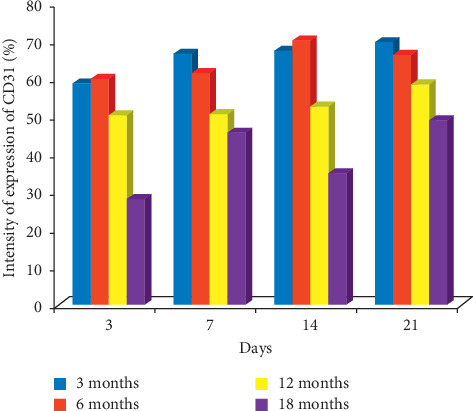
Bar chart indicating the age-related changes in the intensity of expression of CD31 on the gastric mucosa during the healing of gastric ulcer induced with acetic acid.

CD31: the expression of CD31 was higher in 3- and 6-month-old rats than 12- and 18-month-old rats on days 3, 7, 14, and 21 after ulcer induction. On day 14, the labelling index of 70.3%, 67.5%, 52.8%, and 35.3% was obtained for 6-, 3-, 12-, and 18-month-old rats, respectively. On day 21, the labelling index of 69.9%, 66.5%, 58.6%, and 49.0% was obtained for 3-, 6-, 12-, and 18-month-old rats, respectively, which also suggests that the highest expression of factor 8 was seen in 3- and 6-month-old rats during healing as shown in Figures 9 and 10.

## 4. Discussion

The results obtained revealed an inverse relationship between age and rate of ulcer healing. Acetic acid ulcer model used in this study produced ulcers of consistent size and severity at an incidence of 100% and resembled human gastric ulcer in both pathological features and healing mechanism. The slower rate of healing observed in older rats from a macroscopic evaluation of ulcers is consistent with the report from previous work [23].

Healing of gastric ulcer requires reepithelialization as well as potent cell proliferation to fill up mucosal defect and restore gastric glands with the scar [3, 24, 25]. This form of healing is via the proliferation of cells from the ulcer margin and their migration onto the granulation tissue to cover the ulcer base. The poorly differentiated cells from the ulcer margin base sprout into the granulation tissue forming tubules, which transform gastric glands [26, 27].

The protein Ki67 is a biomarker of proliferative activity, which plays a role in ulcer healing; maintaining a dynamic balance between epithelial cell proliferation and apoptosis is essential for maintaining healthy mucosal integrity [28]. Alteration in the balance of epithelial cells proliferation and apoptosis contributes to the formation of gastric ulcer or even carcinogenesis [29, 30]. Therefore, cell proliferation plays a significant role in the healing of gastric ulcers [2, 31]. In this study, Ki67 increased among rats of younger ages (3 and 6 months old) compared with the older rats (12 and 18 months old) during the healing of the acetic acid-induced gastric ulcer. These findings revealed that younger animals have more proliferative activity than older ones during spontaneous healing of the gastric ulcer. Majumdar et al. [13] reported a lower gastric mucosal proliferative response to hypertonic saline-induced acute gastric injury in older rats compared with the younger ones. Subsequent mechanistic studies by Figiel et al. [14] and Relan et al. [15] found out that ageing may be related to diminishing healing capacity of the gastric mucosa that has been damaged by hypertonic saline. Also, this age-related deficiency in mucosa repair is as a result of reduced expression of various growth factors important in the regulation of proliferation (such as transforming growth factor *α*) and growth factor receptor-related enzymes such as tyrosine kinases in the stomachs of aged rats [14, 15].

Epidermal growth factor and transforming growth factor-alpha initiate the stimulus for increased epithelial cell proliferation in the mucosa of the ulcer margin [30]. Epidermal growth factor mediates its biological effects on target enterocytes by binding to a specific 170 kDa membrane-bound glycoprotein receptor, the EGF receptor (EGFR). During the developmental processes of the fetal and adult gastrointestinal tract, liver, and pancreas.

Epidermal growth factor plays an active role [31]. EGFR binding to its receptor activates the intrinsic tyrosine kinase, giving rise to an elaborate cascade of cellular events, which finally results in the synthesis of DNA and cellular growth [32–35]. Healthy mucosal integrity and functions within the gastrointestinal tract are maintained in part by the proliferative activities of EGF [36]. The mechanism involved in the development and progression of the digestive tract injury may be halted by EGF [37, 38], or EGF may accelerate the repair process [39]. Studies have shown that administration of epidermal growth factor could protect against both acid-dependent gastric assaults [37, 40] and independent acid attacks too [38, 40]; there are records of numerous studies on the role of EGF in gastric ulcer healing [39, 41, 42].

The young and old rats in this study managed EGFR integrity despite the induction of ulcer with acetic acid. However, the three- and six-month-old rats showed increased expression of EGFR compared with the older rats for healing to take place. Nonetheless, previous studies described that the increased expression of EGFR in the epithelial cells of gastric ulcer margins is essential for improving to take place [41]. Also, increased phosphorylation of EGFR resulted in an upsurge of MAP-ERK-1 and ERK-2 kinase phosphorylation levels and actions with more than 440% and 880% folds, respectively, at the initial phases of the healing of an experimental gastric ulcer accelerated healing [43]. An increased EGFR expression indicates a vital role of EGF in ulcer healing and scar formation [41, 44].

The development of granulation tissue at ulcer base, new blood vessels formation otherwise called angiogenesis, and restitution of the full glandular architecture of the damaged mucosa may take a few weeks [45]. Angiogenesis is a requirement for the repair of recurring gastroduodenal ulcers [23, 46] through the formation a capillary network; angiogenesis in granulation tissue supplies nutrient and oxygen to the base of ulcer thereby facilitating the process of healing [24, 25, 47]. Angiogenesis is essential in the improvement of gastric mucosa healing and prevention of ulcer relapse [24].

Factor VIII is a blood-clotting protein produced by the liver and some epithelial cells; its activation is in response to cellular injury and malignancy [48]. Ulcer base angiogenesis is an essential factor in ulcer healing; several factors are responsible for accelerated angiogenesis which promote healing, and one of such consideration is factor VIII [49, 50]. In this study, we considered the healing of gastric ulcers induced by acetic acid. There was an increase in the expression of factor VIII in younger rats compared with the older ones, which suggests that the delayed healing seen in old age may be associated with decreased angiogenesis. The result aligns with Amagase et al. [51] reports of reduced angiogenesis in unhealed gastric ulcer.

Platelets endothelial cell adhesion molecule (PECAM-1), also known as cluster of differentiation 31 (CD31), is a protein molecule that is involved in leukocyte migration, angiogenesis, and integrin activation [52]. The secretion is by endothelial cells and some epithelial cell and plays a significant role in removing aged neutrophil from the body [52, 53]. Angiogenesis starts with basement membrane proteolysis [54]. Proteolysis is essential in the induction of microvascular endothelial cell invasion and tube formation. The secretion of proteases requires the activation of both endothelial cells and lymphocytes or monocytes [54].

Their activation requires adhesion molecules interaction [55]. Following proteolysis, tube formation occurs, and a series of adhesion molecules come to play a role in tube formation also. Platelet endothelial cell adhesion molecule (PECAM-1/PECAM-1) interplay is responsible for early events of tube formation [55], which results in a tube-like structure formation as a consequence of cell to cell contact. Therefore, antibodies directed against PECAM-1 can inhibit tube formation in vitro [55].

These results showed that there was an overall gradual increase in the activation of angiogenesis by increasing CD31 expression towards healing and CD31 expression decreased with advancing age after induction of ulcer with acetic acid. The role of the formation of new blood vessels in gastroduodenal ulcer healing and its stimulation in granulation tissues significantly accelerate the healing of experimental duodenal ulcer in rats [56]. Also, Skopiñski et al. [57] reported the age-dependence of human serum angiogenic activity. According to Hoenig et al. [58], ageing is related to endothelial dysfunction as well as decreased progenitor cells (EPC) function and mobilization. In this study, higher angiogenic activities occurred in younger rats compared with older ones, which may be due to activation of innate immunity. The activation of a natural immune response is among the first lines of defense after tissue injury [59]. Restoration of blood flow to the site of injured tissue is a prerequisite for mounting an initial immune response to pathogens and for resultant initiation of a successful repair of wounded tissue [59]. Therefore, the innate immunity of younger rats is more potent than that of the older rats, which encouraged increased angiogenic activities in the younger ones and is responsible for the faster healing rate.

## 5. Conclusion

The study shows that the mechanism underlying the slower rate of healing of gastric ulcer with advancing age in rats may be associated with reduced epithelial cell proliferation and angiogenic activities.

## Figures and Tables

**Table 1:** Changes in the ulcer area after induction with acetic acid in rats of different ages.

	3 months	6 months	12 months	18 months
Day 3 (mm^2^)	6.50 ± 0.21^a^	6.70 ± 0.11^a^	9.08 ± 0.11^b^	9.68 ± 0.27^c^
Day 7 (mm^2^)	5.26 ± 0.09^a^	5.82 ± 0.07^b^	8.00 ± 0.06^c^	8.64 ± 0.19^d^
Day 14 (mm^2^)	1.00 ± 0.22^a^	2.84 ± 0.27^b^	6.32 ± 0.15^c^	6.80 ± 0.06^d^
Day 21 (mm^2^)	0.00 ± 0.00^a^	0.40 ± 0.19^b^	2.20 ± 0.12^c^	2.92 ± 0.40^d^

Different letters show that figures are statistically different at *p* < 0.05.

## Data Availability

The qualitative and quantitative data used to support the findings of this study are included in the article.
